# The role of the hotel industry in the response to emerging epidemics: a case study of SARS in 2003 and H1N1 swine flu in 2009 in Hong Kong

**DOI:** 10.1186/s12992-018-0438-6

**Published:** 2018-11-27

**Authors:** Kevin K. C. Hung, Carman K. M. Mark, May P. S. Yeung, Emily Y. Y. Chan, Colin A. Graham

**Affiliations:** 1 Accident and Emergency Medicine Academic Unit, The Chinese University of Hong Kong, Trauma & Emergency Centre, Prince of Wales Hospital, Shatin, New Territories Hong Kong; 2 JC School of Public Health and Primary Care, The Chinese University of Hong Kong, Prince of Wales Hospital, Shatin, New Territories Hong Kong

**Keywords:** Epidemics, Hotel industry, Infection control, International travel, Private sector engagement, Quarantine, Tourism, Health-related emergency disaster risk management

## Abstract

**Background:**

The global travel and tourism industry has been rapidly expanding in the past decades. The traditional focus on border screening, and by airline and cruise industries may be inadequate due to the incubation period of an infectious disease. This case study highlights the potential role of the hotel industry in epidemic preparedness and response.

**Methods:**

This case study focuses on the epidemic outbreaks of SARS in 2003 and H1N1 swine flu in 2009 in Hong Kong, and the subsequent guidelines published by the health authority in relation to the hotel industry in Hong Kong which provide the backbone for discussion.

**Results:**

The Metropole Hotel hastened the international spread of the 2003 SARS outbreak by the index case infecting visitors from Singapore, Vietnam, Canada as well as local people via close contact with the index case and the environmental contamination. The one-week quarantine of more than 300 guests and staff at the Metropark Hotel during the 2009 H1N1 swine flu exposed gaps in the partnership with the hotel industry. The subsequent guidelines for the hotel industry from the Centre of Health Protection focused largely on the maintenance of hygiene within the hotel premises.

**Conclusion:**

Positive collaborations may bring about effective preparedness across the health and the tourism sectors for future epidemics. Regular hygiene surveillance at hotel facilities, and developing coordination mechanism for impending epidemics on the use of screening, swift reporting and isolation of infected persons may help mitigate the impact of future events. Preparedness and contingency plans for infectious disease control for the hotel industry requires continuous engagement and dialogue.

## Background

The global travel and tourism industry has expanded rapidly in recent years. The global number of international tourist arrivals increased from approximately 541 million in 1995 to 1161 million in 2014 [1]. The ever greater numbers present enormous challenges to the entire global community for epidemic preparedness and control. The increasing complexity of frequent international travel opens an ideal route for local outbreaks of infectious disease to becoming global pandemics.

The health and wellbeing of travellers warrants appropriate consultation and treatment in its own right, but in the case of infectious diseases of major public health concern, it is important to address the public health aspects of their illness as patients are also disease carriers promoting the spread of infectious disease on a potentially global scale. The International Health Regulations (IHR) (2005) form one of the few legally binding instruments of the World Health Organisation (WHO). The purpose and the scope of IHR (2005) are “to prevent, protect against, control and provide a public health response to the international spread of disease in ways that are commensurate with and restricted to public health risks, and which avoid unnecessary interference with international traffic and trade” [2].

Most of the existing research in travel medicine and the current guidelines for international health authorities emphasise the role of the air-travel industry in tracking and thus containing the potential international spread of infectious disease. For instance, the WHO guideline on International Travel and Health highlights the role of airlines as well as shipping companies, together with that of tour operators and travel agents in limiting the spread of infectious disease across borders [3]. Similarly, the Centres for Disease Control and Prevention (CDC) guidelines focus on the airline and cruise industries [4]. The role of the other important sector in tourism, namely the hotel industry, have been less clearly defined and discussed.

This case study will use the example of the Metropole Hotel in Hong Kong in the international spread of Severe Acute Respiratory Syndrome (SARS) in 2003, and the effect of the government mandated quarantine of the Metropark Hotel during the swine flu 2009 in Hong Kong.

SARS was caused by the SARS-associated coronavirus, with the primary mode of transmission being direct mucous membrane contact with infectious respiratory droplets [5, 6]. It had a basic reproduction number of approximately 3, and the spread was mainly to those with close contact in health care and household settings. The first cases were identified in Guangdong province of China in November 2002, and a number of natural reservoirs have been found including civet cats and bats. Incubation period ranged from 3 to 10 days [7]. The overall case fatality ratio was 9.6% worldwide, with deaths resulting from pneumonia and subsequent respiratory failure. The travel industry contributed to the speed of the spread of this unknown disease at that time, particularly within the Metropole Hotel as it was the first site for global dissemination of the virus [8]. SARS subsequently caused 774 deaths in 26 countries, with the disease spreading to cover five continents [7]. This case vividly illustrates how a local outbreak can rapidly evolve into a global pandemic.

The H1N1 swine flu pandemic in 2009 was caused by the A(H1N1) pdm09 influenza virus [9]. This strain containing genes from pig, bird and human influenza viruses has never been reported before 2009, and the outbreak was first identified in March 2009 in Mexico and the United States. It was estimated that between 151,700 and 575,400 deaths resulted globally, with 80% in people younger than 65 years old from respiratory and cardiovascular influenza related complications. The 2009 H1N1 virus infection mortality was estimated to be 0.001–0.011% of the world’s population, much lower than the previous 0.03% during the 1968 pandemic and the 1–3% of the 1918 pandemic [10]. The first imported case in Hong Kong was tested positive for swine influenza on the 1 May 2009 [11]. This has led to the subsequent quarantine of all guests and staff at the Metropark Hotel for 1 week.

Hotels can be a critical component in the evolution of a local outbreak into a global pandemic, and an initial contact point of the import of an impending global pandemic. The aim of this paper is to highlight the role of hotel in the spread of epidemic, and discuss control measures that can be implemented.

## Methods

This case study focuses on the epidemic outbreaks of SARS in 2003 and H1N1 swine flu in 2009 in Hong Kong. Secondary information was extracted from the literature search and the grey literature looking for published official reports, statements, policy papers, field reports and guidelines for further discussion on the role of the hotel industry on the epidemics. Public health principles of disaster response were used to provide the backbone for discussion.

MEDLINE was used to identify research articles published between 1 January 2000 and 31 December 2017 in the English language, to answer these specific questions: 1. what factors in the hotel setting contributed to international spread of SARS; 2. the decision making and implementation of hotel quarantine during swine flu, and the impact of the hotel quarantine.

Any study design regarding aspects on timelines of sequence of events, environmental sampling and contamination, evaluation of hotel related interventions, modelling of interventions were included. Studies not meeting the above inclusion criteria or answering the research questions was excluded.

The search for grey literature involved the searching and browsing the websites of relevant organisations including the World Health Organisation, US CDC, EU European Centre for Disease Prevention and Control, Hong Kong Centre for Health Protection and Department of Health, Food and Health Bureau of Hong Kong SAR government, SARS expert committee, Hong Kong Government information page.

MEDLINE (Ovid SP) search was performed using these terms: (Disease outbreaks [mesh] OR Epidemics [mesh] OR Pandemics [mesh] OR Severe Acute Respiratory Syndrome [mesh] OR Influenza A Virus, H1N1 Subtype [mesh]) AND Hotel [keyword].

## Results

The search identified 34 records from MEDLINE of which five were relevant (references [6, 12–15]), and an additional 13 records were identified through other sources and the grey literature.

### SARS 2003

A medical professor from Guangzhou in China arrived in Hong Kong on 21 February 2003 and checked into a room on the ninth floor of the Metropole Hotel in Kowloon [16]. During his stay, he infected at least seven other guests and visitors staying on the ninth floor of the hotel including three visitors from Singapore, one visitor from Vietnam, two visitors from Canada and a local individual [17].

On 8 March 2003, the Singapore Ministry of Health reported to the Hong Kong Department of Health that three patients who presented with pneumonia were admitted to hospital after returning to Singapore from Hong Kong. They had all stayed in the Metropole Hotel. During the conversation, laboratory investigations were pending and there was not sufficient evidence to suggest that their illnesses had been related to the Metropole Hotel [16].

On 12 March 2003, the WHO issued a global alert about unusual cases of an acute respiratory syndrome. On 14 March 2003, the index case for the significant outbreak at the Prince of Wales Hospital was identified. It was not until 19 March 2003, after multiple enquiries, the patient revealed that he had visited the Metropole Hotel around that period as a visitor but not a guest [16]. On the same day, the Hong Kong Department of Health reported the chain of transmission of the outbreak at the Metropole Hotel [16].

The Metropole Hotel exemplified the potential international spread of infectious diseases. The index cases in the Hong Kong, Toronto, Singapore and Hanoi outbreaks were all associated with the hotel. SARS patients in Ireland and United States had also visited the Metropole Hotel around the same time when there were other sick guests present in the hotel [12–14, 18]. Subsequently there were hospital outbreaks when these index cases returned and were treated at their home countries. Table 1 showed the timeline of the 2003 SARS action of the Department of Health.

**Table 1:** Hong Kong Health Authority’s action to hotel industry during SARS 2003

Date	Action by the Health Authority
21 February 2003	A medical professor from Guangzhou in China arrived in Hong Kong and checked into a room on the ninth floor of the Metropole Hotel in Kowloon. He was later found to be the source of SARS outbreak in Hotel M and beyond.
14 March 2003	The Secretary for Health, Welfare and Food sets up and chairs the 1st meeting of Task Force on SARS.
19 March 2003	Department of Health (DH) announced the chain of transmission of at least 7 SARS cases were related to the Hotel M, the index case of the epidemic in Hong Kong.
19 and 20 March 2003	DH’s Kowloon Regional Office conducted site investigation at Hotel M. DH ensured hotel management conducted proper cleansing and disinfection.
22 March 2003	DH inspected hotel environment inspected and found satisfactory and informed hotel management that they could resume business on 9/F.
28 May and 6 June 2003	DH met with tourism and hotel industries, Tourism Commission and Tourism Board on health awareness programme for visitors.

Note: Materials extracted from references [16, 35]

Little was known about the new disease SARS when the outbreak began at hotel and hospitals in February and early March. The WHO did not issue its first emergency travel advisory naming the illness as SARS until 15 March 2003 [16]. There was local media coverage of an outbreak of atypical pneumonia in Guangzhou on 10 February 2003, and the health authority in Hong Kong had made contact with the Guangzhou and Guangdong authorities. However, accurate information about the atypical pneumonia outbreak in Guangdong Province was not available to Hong Kong or the international community at the time. Case investigation and contact tracing conducted by the Department of Health on 24 February 2003 on the index case revealed that the Guangzhou professor and his wife had stayed at the Metropole Hotel. However, no contact tracing was conducted in the hotel at that stage since the Department of Health had not received any other reports of severe community-acquired pneumonia related to the hotel [16]. There were no environmental factors or triggers identified that warranted further action. However, environmental sampling on the carpet outside the room in which the index case resided, and elevator area showed a hot zone which tested positive for the SARS virus by polymerase chain reaction (PCR) 3 months after the index case stayed at the hotel [19]. Another study investigating German guests staying at the hotel also suggested the possibility of environmental contamination as a source of infection [15]. It is not known how long the infectious virus persists in the surroundings of a SARS patient. The established practice was for contact tracing to be conducted on close contacts (friends or family), but not on the basis of a shared location [16].

A number of researchers estimated the basic reproduction number of SARS by fitting models to the initial growth of the epidemics in a number of countries [19]. Modelling of SARS epidemiology in Hong Kong and China showed rapid public health measures such as contact tracing for confirmed and suspected cases, and quarantining, monitoring, and restricting the travel of contacts had an effective reduction in reproduction number [20–23].

### Swine flu 2009

A 25-year-old male from Mexico arrived in Hong Kong on 30 April 2009, and stayed at the Metropark Hotel. He attended hospital on the same evening where he was admitted to an isolation ward. He subsequently developed a fever and was confirmed to have swine flu on 1 May 2009 [11].

The Hong Kong Special Administrative Region (HKSAR) government raised the response level to ‘Emergency’ on the same day under the *Emergency Preparedness Plan for Influenza Pandemic*. An ‘Emergency Response Level Steering Committee on Human Swine Influenza (Flu A H1N1) Pandemic’ was also established on the 1st May to formulate the overall disease control strategy [11].

Under the *Prevention and Control of Disease Ordinance*, the Director of Health ordered that all guests and staff at the Metropark Hotel should be quarantined on the evening of 1 May 2009 [11]. The quarantine was led by the Department of Health in collaboration with other government departments. Quarantined persons were provided with oseltamivir (Tamiflu) and other medical treatment. The Social Welfare Department provided daily necessities and emotional support to the quarantined. A help desk was set up at the hotel involving the Department of Health, the Home Affairs Department, the Social Welfare Department, the Immigration Department, the Civil Aid Service, the Auxiliary Medical Service and the Police.

The quarantine ended 1 week later, which covered the incubation period of influenza of 1 to 7 days. For those persons who had completed the quarantine period without showing symptoms of being infected, they were issued with Certificates of Conclusion of Quarantine.

At the same time, the Centre for Health Protection (CHP), other government departments and relevant agencies conducted contact tracing starting on the 1st May 2019. Close and selected social contacts were prescribed chemoprophylaxis and put under medical surveillance. The hotel and nearby streets, as well as other public places, were cleansed and disinfected. Hygiene guidelines had been issued to all licensed hotels/guesthouses and rented rooms to encourage enhanced cleansing and improvement of hygiene. All industrial associations had been informed of the situation and reminded the need to take precautionary measures. The Occupational Safety and Health Council had organised public seminars to raise public awareness of preparedness for influenza in the workplace [11]. Table 2 showed the timeline of the 2009 swine flu action of the Department of Health.

**Table 2:** Hong Kong Health Authority’s action to hotel industry during Swine flu 2009

Date	Action by the Health Authority
30 April 2009	A 25-year-old male from Mexico (index patient) arrived in Hong Kong and stayed at the Metropark Hotel.
1 to 7 May 2009	The Director of Health ordered that the Metropark Hotel should be isolated. All guests and staff were quarantined. Quarantined persons were provided with oseltamivir (Tamiflu) and other medical treatment.
1 May 2009	Department of Health conducted contact tracing in the disease containment phase
1 May 2009	Cleansing and disinfection of the hotel lobby and common areas as well as bathrooms in individual guest rooms have been arranged by Food and Environmental Hygiene Department (FEHD).
Early May 2009	The Metropark Hotel as well as 8 other hotels/hostels in which passengers of flight MU505 (flight of the index patient) had stayed have been disinfected.
Early May 2009	Hygiene guidelines have been issued to all owners’ corporations, owners’ committee, mutual assistance committees and the Hong Kong Association of Property Management Companies, licensed hotels/guesthouses and bed space apartments to encourage enhanced cleansing and improvement of hygiene.

Note: Materials taken from references [11, 36]

### Characteristics of hotels involved

Both the Metropole Hotel at Kowloon (now renamed Metropark Hotel Kowloon) and the Metropark Hotel at Wan Chai (now renamed Kew Green Hotel) were four stars hotels situated at busy part of the city. Both hotels were managed by the same management group – the China Travel Service (Holdings) Hong Kong Limited. The two hotels were no different in terms of access to public health facilities and general standard of care. The difference in the timing of public health actions by the health authority was likely contributed by experience of SARS preceding swine flu.

### Government initiatives on epidemic preparedness with the hotel industry after SARS and swine flu

After the SARS outbreak in Hong Kong the health authority established the Guidelines for Hotels in Preventing Severe Acute Respiratory Syndrome (SARS) [24] and Guidelines on Infection Control & Prevention in the Hotel Industry [25]. The guidelines provided practical information for hotel staff members on how measures to prevent communicable diseases should be done. It offered comprehensive information on ways to implement infection control measures, in particular the maintenance of good hygiene on hotel premises [25].

The CHP organised Infection Control Seminars for the Hotel Industry on a regular basis. For instance, in response to the Ebola virus outbreak in West Africa in 2014–2016, the CHP provided advice for the local hotel industry on receiving guests with a travel history or residence in an Ebola virus disease affected area. The guideline stressed the importance of enquiring about the travel history of guests and outlined procedures on handling these guests who may feel unwell. The guideline reiterated the need to keep a record of staff and residents who had stayed in the hotel, with their personal and contact details, for possible future public health actions and contact tracing [26].

Ideally, hotels should be setting their own standards of hygiene measures and providing training to staff before an outbreak occurs. Further roles and responsibilities included contingency arrangement, plan of acquisition of protective equipment, disease reporting and surveillance mechanism during outbreak period [27].

From our online and database internet search, however, there is little mention of collaboration between the government and the hotel industry. No documentation was found on setting up of task forces or committees, or of invitation to hotel representatives to the working group advising on infection control guidelines in Hong Kong.

## Discussion

SARS served as the classic example of how tourism and international travel can present challenges to the global health system. The spread of the illness within a single hotel and the subsequent international air travel of the victims contributed to and accelerated the speed of the spread of SARS across the globe.

The experience from SARS in Hong Kong had a profound impact on the public health reform especially on the infectious disease surveillance and epidemic response [16]. These included strengthening the surveillance and the isolation and treatment of individuals with the disease according to case definitions, the establishment of communication channels between hotel and the government system, and the development of guidelines and response plans that allows the implementation of stringent infection control measures when necessary. The establishment of contingency plans and command structures including the ‘Emergency Response Level Steering Committee on Influenza Pandemic’ allowed a clear structural framework and key lines of responsibility [28]. However, collaboration with the private sector and the hotel industry were found to be limited and focused around infection control measures.

According to subsequently published literature, application of appropriate measures had likely reduced the number of people who were infected, requiring medical care and died during the influenza pandemic [29]. It has been shown that case isolation or household quarantine could have a significant impact at reducing attack rates in the community, and chemoprophylaxis can greatly reduce disease transmission during the pandemic [30, 31]. However, the quarantine of guests at the Metropark Hotel in 2009 inevitably stirs up much discussion and controversy among the media and the public health community on the balance between the need to protect the public health and the need to safeguard civil liberties.

The decision of quarantine created enormous tension between the government, guests and the hotel management. The decision of the need for quarantine and the scale of the quarantine needs to be scientifically justified. The negative effects overall of such a policy on the tourism attractiveness of a destination cannot be neglected. The quarantine at the Metropark Hotel during swine flu also highlighted the extensive assistance needed for the quarantined persons, and the cooperation necessary in the possible future need for a hotel quarantine. Pre-established partnerships and coordination between the government and the hotel industry is key in epidemic preparedness and response.

### Effects of epidemics on the hotel industry

Studies have shown that the psychological impacts of SARS and the government restrictions on travel, had a great impact on the travel industry far beyond the region of SARS hit areas [32]. For the hotel industry in Hong Kong, the number of hotel guests dropped dramatically to a level that was never experienced before [33]. In order for hotels to sustain their business, the Hong Kong hotel industry adopted an industry-wide recovery effort and empathized on mutual support [26]. Previous papers called for a better preparedness of the hotel industry for future crises and epidemics [32, 33].

### Collaborations with the hotel industry to mitigate the impact of epidemics

Hotels are often the first point of contact for tourists arriving at a host country. Hotels could provide an additional line of defence beyond entry border screening, and they could offer another layer of protection against illnesses that border screening processes may have missed, for example in the situation where travel occurs during the incubation period of an infectious disease. In view of this, the capacity of hotels in the detection of potential illness and the launching of an initial response should be fully recognised and utilised.

The WHO pandemic influenza risk management recommended involving civil society and the private business sector in pandemic preparedness planning and national committees [34]. The case of hotel industry collaboration with the health sector in Hong Kong has the potential to provide a positive example of effective disaster risk reduction coordination.

## Conclusions

The epidemic preparedness and infection control measures mounted against SARS and H1N1 swine flu demonstrated a role that needed to be filled by the hotel industry. During SARS, late recognition of the environmental contamination of hotel facilities and the failure of timely intervention on the hotel guests with close contact contributed to the spread of the disease internationally. While the appropriateness and best method of quarantine in future pandemic influenza warrants further research, the 2009 swine flu hotel quarantine exposed gaps in the partnership with hotel industry. Health authorities in Hong Kong had since provided guidelines mostly in the area of disinfection and hygiene, and focused on educating hotel workers on basic hygiene to prevent the spread of infectious diseases. The potential to establish traveller screening, timely reporting and isolation for the infected guests during epidemics could be explored. The capacity of the hotel industry in controlling infections should be recognised not only in Hong Kong but also in other parts of the world.
